# Role of Vascular Endothelial Growth Factor (VEGF) in Human Embryo Implantation: Clinical Implications

**DOI:** 10.3390/biom11020253

**Published:** 2021-02-10

**Authors:** Xi Guo, Hong Yi, Tin Chiu Li, Yu Wang, Huilin Wang, Xiaoyan Chen

**Affiliations:** 1 Department of Obstetrics and Gynaecology, Shenzhen Baoan Women’s and Children’s Hospital, Shenzhen University, Shenzhen 518133, China; guoxi@link.cuhk.edu.hk (X.G.); wangyu1631@outlook.com (Y.W.); 2 Department of Obstetrics and Gynaecology, Faculty of Medicine, The Chinese University of Hong Kong, Hong Kong, China; tinchiu.li@cuhk.edu.hk; 3 Department of Reproductive Health, Shenzhen Baoan Women’s and Children’s Hospital, Shenzhen University, Shenzhen 518133, China; yihong188@outlook.com; 4 Department of Central Lab, Shenzhen Baoan Women’s and Children’s Hospital, Shenzhen University, Shenzhen 518133, China

**Keywords:** vascular endothelial growth factor (VEGF), embryo implantation, reproductive failure

## Abstract

Vascular endothelial growth factor (VEGF) is a well-known angiogenic factor that plays a critical role in various physiological and pathological processes. VEGF also contributes to the process of embryo implantation by enhancing embryo development, improving endometrial receptivity, and facilitating the interactions between the developing embryo and the endometrium. There is a correlation between the alteration of VEGF expression and reproductive failure, including recurrent implantation failure (RIF) and recurrent miscarriage (RM). In order to clarify the role of VEGF in embryo implantation, we reviewed recent literature concerning the expression and function of VEGF in the reproductive system around the time of embryo implantation and we provide a summary of the findings reported so far. We also explored the effects and the possible underlying mechanisms of action of VEGF in embryo implantation.

## 1. Introduction

Vascular endothelial growth factor (VEGF) is a multi-functional factor primarily involved in the regulation of proliferation, differentiation and survival of endothelial cells as well as in vascular permeability [1]. The family of VEGF consists of a group of growth proteins including VEGF-A–VEGF-F, placental growth factor (PlGF), and endocrine gland-derived vascular endothelial growth factor (EG-VEGF) [2]. VEGF-A (also called VEGF), which was firstly described by Senger et al. in 1983 [3], has been proved to be the most important and potent factor in angiogenesis [2]. PlGF, on the other hand, is thought to be selectively involved in pathological angiogenesis, for instance, in tumors and in ischemic and inflammatory processes [4,5]. VEGF-B is more involved in the growth, differentiation, and survival of certain types of cells [6,7], while VEGF-C and VEGF-D are primarily implicated in lymphangiogenesis [8,9].

VEGFs exert their effects mainly through binding to tyrosine kinase receptors: fms-like tyrosine kinase 1 (Flt-1, also termed VEGFR-1), kinase insert domain receptor (KDR, also termed VEGFR-2), and Flt-4 (also termed VEGFR-3) [2]. VEGFR-2, which has the strongest pro-angiogenic activity, is mainly expressed in vascular endothelial cells and can bind to VEGF [2]. Compared with VEGFR-1, VEGFR-2 has a higher tyrosine kinase activity but a lower affinity for VEGF [10,11]. Besides the expression in endothelial cells, VEGFR-1 is also expressed in macrophage-lineage cells [11]. VEGFR-1 can interact with VEGF, VEGF-B, and PlGF [12,13]. With a higher affinity but lower kinase activity, VEGFR-1 acts more like a decoy, a negative regulator of VEGF [14,15]. sVEGFR-1 (sFlt-1), a soluble form of VEGFR-1, can also trap VEGF, VEGF-B, and PlGF and therefore block their binding to membrane receptors [16,17], and its activity has been proven to be strongly correlated with unexplained infertility [18,19], recurrent miscarriage [20], and adverse pregnancy outcomes [21]. PlGF, which shows lower affinity for VEGFR-1 than VEGF [22], can replace VEGF in the VEGFR-1 “sink” and thus potentiate the angiogenic effect of VEGF, since VEGFR-2 is the main receptor with a pro-angiogenesis effect [4,12]. VEGFR-3 is expressed in lymphatic endothelia and high endothelial venules [23]. Through binding with VEGF-C or VEGF-D, VEGFR-3 transduces signals for lymphangiogenesis [2]. In addition to these three tyrosine kinase receptors, VEGF can also bind to neuropilins which act as co-receptors [24].

VEGF is implicated in a wide variety of physiological and pathological conditions. During the process of embryo development, VEGF participates in embryonic vasculogenesis and angiogenesis [25,26]. Moreover, in postnatal development, there is accumulating evidence showing the crucial role of VEGF in body growth and organ development [27,28,29]. In inflammation, VEGFR-1 plays a role in the recruitment and activation of monocytes and macrophages [30,31,32]. In oncogenesis, VEGF is responsible for tumor growth and metastasis, based on which anti-angiogenic therapy has achieved great progress in tumor treatment [1]. In the field of reproduction, extensive efforts have been made to clarify the role of VEGF in embryo implantation. However, there is a lack of a comprehensive summary of the existing data. In this review article, we summarized recent literature concerning the expression and function of VEGF in the reproductive system around the time of embryo implantation. We also explored the effects and the possible underlying mechanisms of action of VEGF in embryo implantation.

## 2. The Role of VEGF in the Reproductive System during Embryo Implantation

### 2.1. VEGF in Human Endometrium

Endometrium lines the inside of the uterus and undergoes cyclic breakdown and remodeling, which are accompanied with the reconstruction of the vascular system. During the menstrual cycle, there is only a short and critical period of time allowing an embryo to implant, when the endometrium becomes a well-vascularized tissue characterized by increased vascular permeability, oedema, and angiogenesis [33]. Although a myriad of factors and cytokines are thought to be involved in this transformation, VEGF, as a potent angiogenic factor, plays a central role.

Extensive efforts have been made to explore the spatial and temporal expression change of VEGF in the endometrium across the menstrual cycle. Several studies have found the expression of VEGF in the endometrium throughout the menstrual cycle, with a significant increase in the mid-luteal phase, suggesting a role of endometrial VEGF around the time of embryo implantation [34,35,36]. In addition, adequate expression of VEGF is essential for successful pregnancy, based on previous finding that endometrial VEGF expression is impaired in the peri-implantation period in infertile patients [37,38]. Functional experiments have shown that the addition of VEGF could promote embryo outgrowth as well as the adhesive capacity of endometrial epithelial cells [39]. In women with recurrent miscarriage, VEGF showed a lower expression level in the endometrium during the mid-luteal phase [40]. However, one of our previous studies has identified elevated VEGF levels in women with hampered pregnancy potential [41]. In that study, endometrial tissues were obtained from women with elevated progesterone level. Progesterone and estrogen are two main regulators of endometrium changes across the menstrual cycle and have significant promoting effects on VEGF production [42,43]. Therefore, the analysis of VEGF levels should take into account the effects of estrogen, progesterone, and other potential influential factors to understand the role of VEGF during embryo implantation.

In accordance with the upregulation of VEGF, VEGFR-1 and VEGFR-2 are also upregulated in the glandular epithelium in the secretory phase [44]. sFlt-1, a soluble antagonist of VEGF and PlGF, is increased distinctively in the proliferative phase but decreased to almost 25% of its proliferative phase peak value in the endometrium during the luteal phase [45]. The analysis of serum sFlt-1 level showed that infertile women had higher serum sFlt-1 levels in the secretory phase than in the proliferative phase [19]. Experimentally, sFlt-1 expression in human primary endometrial stromal cells is also found to be turned off during decidualization, whereas VEGF expression showed an opposite change [46]. The upregulation of VEGF and the coordinated downregulation of sFlt-1 in the luteal phase may serve to increase endometrial angiogenesis and vascular permeability, which are crucial for endometrium preparation for embryo implantation.

Histochemistry staining reported that PlGF, which exists in glandular and luminal epithelial cells, decidual stromal cells, and uterine fluid, showed increased staining intensity in the endometrium around the time of embryo implantation [47]. Santi et al. also demonstrated that PlGF expression was higher in the endometrium of women with successful embryo implantation compared to those who failed to conceive [48]. In that study, the authors firstly reported that PlGF gene expression was positively correlated with the hysteroscopic appearance of the endometrium based on the Sakumoto-Masamoto grading system. In contrast, one of our previous studies found that PlGF was upregulated in women with a high serum progesterone level, and this increase might be a cause of impaired receptivity of the endometrium in those women [41]. As described before, PlGF and VEGF are two synergistic molecules acting on angiogenesis. The upregulation of these two cytokines in the endometrium during the time of embryo implantation suggests that angiogenesis is crucial for successful embryo implantation. Moreover, the upregulation of VEGF and its receptors in the secretory phase might aim to keep pace with the rapidly thickening endometrium. Another study also showed that stromal VEGF intensity and stromal microvessel density (MVD) could be positive predictors of pregnancy outcomes in preparation of frozen embryo transfer (FET) cycles [49].

**Figure 1:**
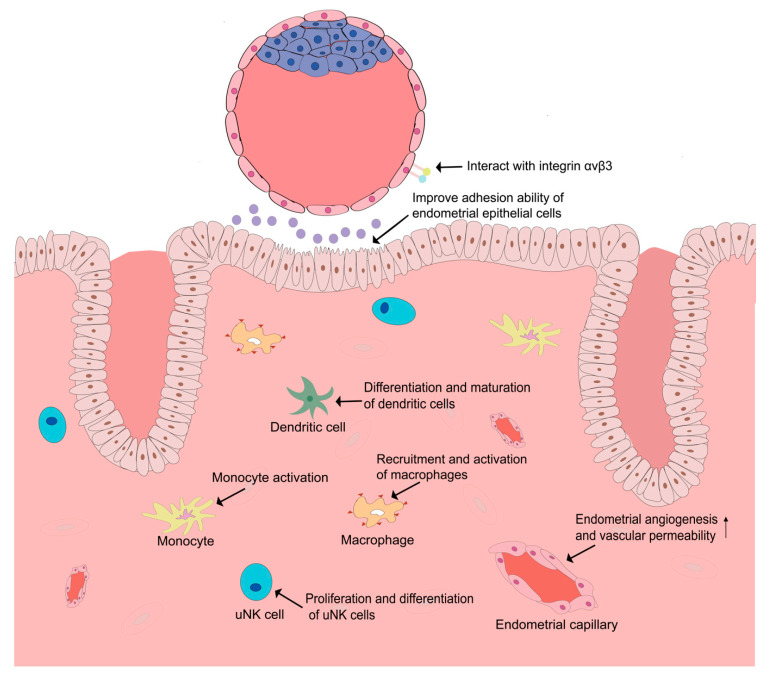
Role of vascular endothelial growth factor (VEGF) in the human endometrium and interaction between endometrium and embryo.

It is worth noting that besides an angiogenic effect, VEGF and PlGF are also functionally related to immune regulation. An embryo is an allograft to the maternal immune system, and immunologic tolerance is critical for the successful establishment of embryo implantation. During the establishment of immuno-tolerance, numerous immunosuppressive mechanisms and various immunocytes, including uterine natural killer (uNK) cells, macrophages, and dendritic cells, are thought to play significant roles [50,51]. Several studies have demonstrated that VEGF and PlGF might serve as immune modulators and mediate the immuno-tolerance of the maternal immune system during the time of embryo implantation. Monocytes, well-characterized immunocytes [52], could be activated by VEGF and PlGF [53,54]. VEGF and PlGF also promote the recruitment and activation of macrophages [32,55,56]. PlGF is also found expressed in uNK cells and plays a significant role in the proliferation and differentiation of uNK cells [57]. PlGF is also implicated in the regulation of the differentiation and maturation of dendritic cells and is able to skew type 1 T helper immune response to the Th2 phenotype [58]. All these immunoregulatory functions of VEGF and PlGF might be favorable for the establishment of pregnancy (Figure 1, Table 1).

### 2.2. VEGF in the Development of Human Oocytes and Embryo

The growth and development of follicles are dependent on an adequate blood supply to obtain nutrients and oxygen. Insufficient blood supply could lead to a low-oxygen status in follicular fluid and severely disrupt the development of oocytes and, subsequently, of the embryo [71]. The inhibition of angiogenesis could lead to disruption of follicular development, ovulation, and endocrine functions of the ovary [72,73]. VEGF is secreted by granulosa cells and theca cells and can be detected in the follicular fluid [74,75]. A high concentration of VEGF in the follicular fluid is correlated with increased perifollicular vascularity, higher fertilization rates, better embryo quality, and higher pregnancy rates [61]. As VEGF in the follicular fluid is mainly secreted from granulosa and theca cells, it is likely that a higher VEGF level indicates better functioning of these cells and thus the presence of a better microenvironment for follicle development. In contrast, there are several studies indicating that VEGF concentration in follicular fluid is negatively correlated with in vitro fertilization (IVF) pregnancy outcomes [76,77]. However, in these studies, women with polycystic ovary syndrome (PCOS) were included. It is well recognized that PCOS patients have a higher level of VEGF and compromised IVF outcomes [78]. The inclusion of PCOS patients could be a remarkable influential factor in these analyses.

**Figure 2:**
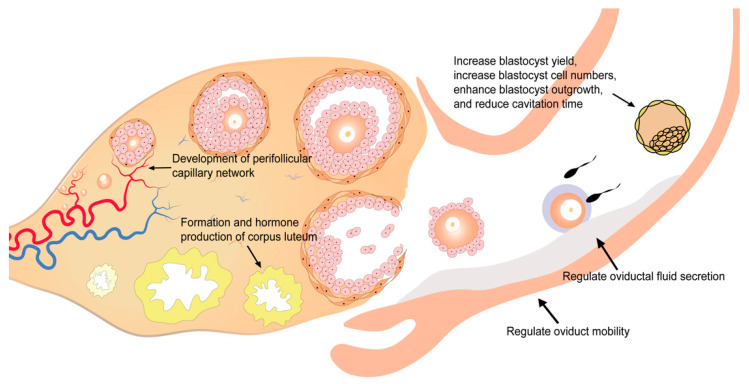
Role of VEGF in the development of human oocytes and embryo.

VEGF also acts in the formation and function of corpus luteum (Table 1, Figure 2). Corpus luteum is a transient endocrine gland that develops from residual follicular tissues after ovulation in the presence of active angiogenesis [79]. The ruptured follicle is under hypoxia conditions after ovulation, when hypoxia-inducible factor-1 (HIF1) is induced and promotes the angiogenesis of corpus luteum via VEGF [62]. Besides the formation of corpus luteum, VEGF also contributes to progesterone production by corpus luteum [63]. The reduction of VEGF and VEGFR-2 levels in corpus luteum is related to the impairment of the luteal circulation [80]. Blocking of VEGF signaling via VEGFR inhibition is proved to induce decreased luteal endothelial networks, vascular endothelial cell detachment, apoptosis of luteal steroid-producing epithelial cells, disrupted luteal function, embryonic development arrest, and preterm birth [63,80,81].

With a highest level during the periovulatory period, VEGF shows dynamic changes in the human oviduct across the menstrual cycle and is thought to be implicated in the secretion of oviductal fluid via regulating the vascular permeability of the oviduct (Table 1, Figure 2) [65,66]. Furthermore, VEGF also plays a role in oviduct mobility (Table 1, Figure 2). VEGF regulates oviduct contraction by stimulating the biosynthesis and release of prostaglandin E2 (PGE2), prostaglandin F2α (PGF2α), and endothelin-1 (ET-1) in bovine oviductal epithelial cells, which is important for gametes transport, fertilization, and embryo transport [64].

VEGF can be detected throughout the process of embryo development [82,83]. The analysis of embryos cultured in vitro also demonstrated that the expression of VEGF and two VEGF receptors (VEGFR-1 and VEGFR-2) was maintained during embryo development [84]. Functional in vitro experiments in embryos suggest that the addition of VEGF could increase blastocyst yield and blastocyst cell numbers, enhance blastocyst outgrowth, and reduce cavitation time [39,67,68]. As another member of the VEGF family, PlGF shows a similar effect on embryo development: increased blastocyst cell numbers and enhanced blastocyst outgrowth [47]. However, the molecular mechanisms underlying these effects remain unclear.

### 2.3. VEGF in the Interaction between Endometrium and Embryos

After an embryo enters the uterus lumen, the interaction between embryo and endometrium starts in the uterine fluid through locally produced soluble mediators. Uterine fluid, also called uterine secretion, is mainly produced by uterine glands. During the peri-implantation period, endometrial glands secrete important mediators that facilitate pre-implantation embryo development and embryo implantation. Several VEGF family members such as VEGF and PlGF have been identified in uterine fluid [39,47]. It is demonstrated that VEGF concentration was upregulated in mid-luteal uterine fluid [39]. The significant role of VEGF in the dialogue between embryo and endometrium is further evidenced by functional experiments where enhanced blastocyst outgrowth, improved endometrial epithelial cell adhesion ability, reduced cavitation time, increased blastocyst cell numbers, increased implantation rates, and enhanced fetal limb development were observed after VEGF treatment [39,67]. PlGF is found upregulated in endometrial glands, with strong corresponding staining on the apical surface of the epithelium in mid-secretory phase [47]. Functional studies of PlGF showed similar results, indicating that PlGF increased blastocyst cell numbers, enhanced blastocyst outgrowth, and improved endometrial epithelial cells’ adhesion ability [47]. In addition to the effect of endometrium-derived VEGFs on the embryo, embryo-derived factors and cytokines are also involved in the regulation of the uterine microenvironment. For example, embryo-derived VEGF-A stimulates endometrial angiogenesis, which enables embryos to induce angiogenesis directly at the implantation site [69]. Human chorionic gonadotropin (hCG), produced by the embryo, is also a stimulator of VEGF action in the endometrium [85]. Taken these results together, although less is known about the effect of the embryo on the endometrium, the crosstalk of embryo and endometrium might be extensive and influence embryo implantation in a more complex manner (Figure 1, Table 1). 

Furthermore, VEGFR-2 could interact with integrin αvβ3 [70], which is an important adhesion molecule during embryo implantation [86], in VEGF-induced angiogenesis. Integrin αvβ3 is found to be expressed in endometrial pinopodes and blastocyst trophectoderm [87], and the ligand of integrin αvβ3, the glycoprotein osteopontin (OPN), is also expressed in the endometrium. The interaction of embryonic integrin αvβ3 and endometrial OPN is thought to be involved in embryo adhesion to the luminal epithelium of the endometrium, while the binding of VEGFR-2 and integrin could shift the cell surface localization of VEGFR-2 to focal adhesions and induce endothelial cell polarization, which could also be a mechanism facilitating embryo implantation [88]. However, further studies are still necessary to explore how this interaction between VEGFR-2 and integrin αvβ3 influences embryo implantation.

## 3. VEGF in Reproductive Failure

### 3.1. Recurrent Implantation Failure (RIF)

RIF occurs when a woman under the age of 40 fails to achieve a clinical pregnancy after transfer of at least four good-quality embryos in a minimum of three fresh or frozen embryo transfer cycles [89]. There are numerous factors that could lead to RIF, such as uterine abnormalities, advanced maternal age, elevated body mass index, immunological factors, and abnormal angiogenesis [90,91].

One earlier study has reported an increased serum VEGF level in women with RIF compared to fertile controls [92], whereas one of our earlier studies demonstrated decreased VEGF expression in all regions of the endometrium at the time of embryo implantation [91]. The discrepancy between these two studies might derive from different sampling time and different sample types. Further studies are needed to explore the expression pattern of VEGF in RIF patients. Furthermore, some studies have explored the association between VEGF polymorphisms and the occurrence of embryo implantation failure. These results suggest polymorphisms of the VEGF gene could impact fertilization rate, embryo implantation rate, and pregnancy rate [93]. In addition, VEGF polymorphisms such as VEGF-1154A/A, which are related to altered VEGF expression, increase the risk of RIF [94,95,96,97]. However, whether VEGF influences embryo implantation in RIF patients remains largely unknown.

### 3.2. Recurrent Miscarriage (RM) 

RM is defined as three or more consecutive miscarriages before 24 weeks of gestation [98]. Although several known causes of RM, including parental chromosomal anomalies, uterine malformation, endocrinological disorders, and immunological abnormality, are known, around half of RM cases remain unexplained [99]. Recent evidence has indicated that altered expression of VEGF family members might be a contributory factor to RM [20,100]. 

Lash et al. explored the expression of VEGF and its receptors in women with unexplained recurrent miscarriage [38]. The results demonstrated that, during mid-late secretory phase, the expression of VEGF-A in vascular smooth muscle cells (VSMCs), endothelial cells (ECs), and glandular epithelial cells was decreased, while the expression of VEGFR-1 in stromal cells, VSMCs, ECs, and glandular epithelial cells was increased in RM patients. This study also showed a decreased expression of VEGFR-2 in VSMCs and stromal cells, an increased expression of VEGFR-3 in glandular epithelial cells, and a greater proportion of mature vessels in the endometrium around the time of embryo implantation in women with RM. Banerjee et al. identified lower VEGF expression in endometrial tissues from patients with idiopathic recurrent miscarriage (IRM) in the peri-implantation period [101], and the observed downregulation of VEGF might be a product of the downregulation of angiogenic cytokines including interleukin (IL)-2, IL-6, and IL-8. In another previous study, Amirchaghmaghi et al. analyzed the gene expression of VEGF, VEGFR-1, and VEGFR-2 and found that unexplained RM (URM) patients had a lower VEGF level and higher VEGFR-1 and VEGFR-2 levels in the endometrium between day 19th and 24th of the menstrual cycle when compared to fertile controls [100]. In contrast, in one of our earlier studies, RM patients were found to have a higher VEGF level in luminal epithelium, glandular epithelium, and stroma of the endometrium around the time of embryo implantation [91]. RM patients were also found to have higher HIF1α expression and increased number of micro-blood vessels in the endometrium [102]. These contradictory results imply the multifactorial nature of the etiology of RM, although all patients were diagnosed with unexplained RM at the time of sampling. 

In addition to the endometrial tissues, there are also some studies focusing on VEGF expression in other types of tissue from women with recurrent miscarriage. By sampling chorionic villi from RM patients, Pang et al. found that VEGF and sFlt-1 levels were increased when compared to their levels in women with a normal pregnancy [103]. However, other studies demonstrated that VEGF expression in the chorionic villi and decidua from women with recurrent pregnancy loss (RPL) was significantly decreased compared with the expression in women with normal pregnancy [104,105], while in peripheral blood, serum VEGF expression was higher in URM patients between day 19th and 24th of the menstrual cycle when compared to VEGF level in fertile controls [100]. In accordance with these results, Pang et al. also found that the serum levels of VEGF and sFlt-1 in RM women were significantly higher than in women with normal pregnancy [20]. However, several studies reported opposite results, though the time of serum sampling was not mentioned [106,107,108]. Therefore, given the significantly different changes during the menstrual cycle and pregnancy, it is crucial to standardize the timing of sampling in future studies.

Although there is compelling evidence showing dysregulation of VEGF in a number of common diseases, the underlying causes of this dysregulation remain unknown. Genetic variation might be one of the potential causes responsible for dysregulated VEGF expression [109]. A meta-analysis of 10 independent case–control studies revealed that rs1570360, rs3025039, rs2010963, and rs3025020 polymorphisms of VEGF were associated with elevated RM risk [110]. Furthermore, Su et al. reported that KDR polymorphisms were correlated with RPL [111]. Hence, further studies are needed to illustrate the mechanisms by which VEGF is regulated in RM.

### 3.3. Endometriosis

Endometriosis is a common cause of infertility in women of reproductive age. There is accumulating evidence showing that eutopic endometrium in women with endometriosis displays higher VEGF expression [112,113]. At the same time, estrogen receptor is also upregulated in eutopic endometrium of patients with endometriosis [112]. Thus, the endometrium of endometriosis patients might be hyper-responsive to estrogen stimulation and thus enhance the expression of VEGF. In addition, the dysregulation of VEGFRs (downregulation of VEGFR-1 and upregulation of VEGFR-2) seems to be responsible for endometriosis [114]. Kim et al. have shown that expression of VEGFR-1, VEGFR-2, and VEGFR-3 was higher in the mid-luteal endometrium of patients with endometriosis [115]. The authors also conducted a subgroup analysis and found that in patients with endometriosis, non-pregnant subjects had higher VEGFR-1 and VEGFR-3 expression than pregnant subjects [115]. Moreover, endometriotic mesenchymal stem cells of ectopic lesions from endometriosis patients exhibited unique biological characteristics, with increased production of angiogenic factors including VEGF and platelet-derived growth factor (PDGF), which implies intrinsic defects in these cells [116]. In addition, anti-VEGF therapy could attenuate the progress of endometriosis and may be a novel strategy for endometriosis treatment [117]. All these findings suggest the important role of VEGF in endometriosis. 

### 3.4. PCOS

PCOS is a common endocrine disease characterized by oligomenorrhea or amenorrhea, hyperandrogenism, and the presence of polycystic ovary. In addition to endocrine disorders, ovarian hyperplasia and hypervascularity are also two common features of PCOS and are thought to be related to extensive angiogenesis of the ovary [118,119]. These angiogenic disorders might be associated with the decreased ovulation rates of PCOS patients [120]. Immunohistochemistry analysis found increased VEGF expression in PCOS ovary [121], which might be correlated with increased vascularity in the ovarian stroma and the higher incidence of ovarian hyperstimulation syndrome (OHSS) observed in PCOS patients [122,123,124]. After VEGF inhibitor treatment, the ovary from PCOS rats demonstrated a decreased percentage of primary follicles and improved ovulation and follicular development [125].

It has been proved that VEGF levels in serum and follicular fluid of PCOS patients were significantly higher, while sFlt-1 level was lower than in normal ovulatory women [78,126]. Taken these results together, the upregulation of VEGF and the downregulation of VEGF antagonists may jointly increase VEGF bioavailability in PCOS patients. However, in PCOS patients, the expression of VEGF in the endometrium around the time of embryo implantation is lower than in controls [127]. As VEGF is upregulated in the mid-luteal phase in normal endometrium as described above, the downregulation of VEGF might be a contributory factor to impaired endometrial receptivity in patients with PCOS. Moreover, VEGF polymorphisms are also found to be related to PCOS [128,129].

### 3.5. Preeclampsia (PE)

PE is a serious pregnancy complication characterized with hypertension and proteinuria. A defect in placental angiogenesis is considered to be one of the factors responsible for the pathogenesis of PE [130]. Significant attention has been given to the alteration of the VEGF system in PE, since it is the main regulator of placental angiogenesis. 

sFlt-1 was found to be upregulated [131,132,133,134], whilst VEGF and PlGF were downregulated in serum and placenta of PE patients [131,132,135,136]. VEGFR-1 and VEGFR-2 were found to be overexpressed in the placenta from patients suffering from PE [130]. Placentation is a process with extensive angiogenesis in order to establish an appropriate vascular network between mother and fetus [137,138,139]. VEGF mediates angiogenesis and has an anti-apoptotic effect on vascular endothelium cells [2]. VEGF also plays a role in the proliferation, migration, and endovascular differentiation of trophoblast cells [140,141]. Thus, the alteration of the VEGF system could lead to placental malfunction. Maynard et al. have elucidated the central role of sFlt-1 in the pathogenesis of PE [133]. At an early stage of pregnancy, hypoxia increases sFlt-1 production by placental cytotrophoblasts [142]. Subsequently, excess sFlt-1 produced by the placenta leads to endothelial dysfunction, hypertension, and proteinuria by trapping VEGF and PlGF [133]. Thus, among these changes in the VEGF system, the elevation of sFlt-1 might play a central role, since decreased concentrations of VEGF and PlGF might be a consequence of an elevated level of circulating sFlt-1. 

Based on the fact that the process of angiogenesis is regulated by numerous pro-angiogenic and anti-angiogenic factors and cytokines as well as angiogenic receptors, there is an increasing number of studies focusing on the balance between pro-angiogenic and anti-angiogenic factors in preeclampsia [143,144]. In some studies, the ratios sFlt-1/PlGF and VEGF/ sFlt-1 were determined to study the association of angiogenesis with certain pathological conditions [19,21,145].

### 3.6. Anti-Angiogenic Therapy

Over these years, the development of anti-VEGF agents has achieved great progress [146,147]. A number of anti-angiogenic agents including bevacizumab have been applied clinically in tumor treatment [1]. Associated with conventional chemotherapy, bevacizumab significantly improved the prognosis of patients with metastatic colorectal cancer or non-small-cell lung cancer [148,149,150,151,152]. Anti-angiogenic therapy is also proved to be effective in the treatment of non-tumor diseases, for instance, age-related macular degeneration [153]. However, as stated above, due to the complex role of VEGF in the reproductive system, anti-VEGF therapy has not been applied in clinical practice in the field of reproductive medicine yet. Although some studies have reported the possible therapeutic role of VEGF inhibitors in women with PCOS [125], the clinical application of anti-VEGF therapy should be cautious before we fully understand the underlying mechanism whereby VEGF influences embryo implantation and the pharmacokinetic characteristics of these drugs.

## 4. Conclusions

VEGF is an important angiogenic factor in many physiological and pathological conditions. In this review, we summarized recent data on the role of VEGF in embryo implantation and reproductive failure. The existing data show that VEGF plays multifaceted roles in embryo implantation and that the alteration of the expression of VEGF, including VEGF polymorphisms, could lead to infertility and pregnancy complications. However, little is known about the mechanisms whereby VEGF influences embryo implantation or the root causes of the observed alterations in VEGF expression. Further studies are in urgent need to clarify the role of VEGF in successful pregnancy and reproductive failure. 

## Figures and Tables

**Table 1:** Role of VEGF in embryo implantation. PlGF, placental growth factor, VEGFR-2, VEGF receptor-2.

VEGF Family Members	Function
Endometrial receptivity	
VEGF	Promotes endometrial angiogenesis and vascular permeability [59,60]
VEGF	Monocyte activation [54]
VEGF	Recruitment and activation of macrophages [32,56]
PlGF	Monocyte activation [53]
PlGF	Recruitment and activation of macrophages [55]
PlGF	Proliferation and differentiation of uNK cells [57]
PlGF	Differentiation and maturation of dendritic cells; skews type 1 T helper immune response to the Th2 phenotype [58]
Embryo development	
VEGF	Development of the perifollicular capillary network [61].
VEGF	Formation of and hormone production by corpus luteum [62,63]
VEGF	Regulates oviduct mobility [64]
VEGF	Regulates oviductal fluid secretion [65,66]
VEGF	Increases blastocyst yield and blastocyst cell numbers, enhances blastocyst outgrowth, and reduces cavitation time [39,67,68]
PlGF	Increases blastocyst cell numbers and enhances blastocyst outgrowth [47]
Embryo implantation	
VEGF	Improves endometrial epithelial cells’ adhesion ability and increases implantation rates [39,67]
VEGF	Embryo-derived VEGF stimulates angiogenesis at the implantation site [69]
VEGFR-2	Interacts with integrin αvβ3 [70]
PlGF	Improves endometrial epithelial cells’ adhesion ability [47]
